# A Remarkable Explosion

**Published:** 1885-01

**Authors:** 


### A REMARKABLE EXPLOSION.

One of the most remarkable explosions in the history of the oil region occurred at Scrubgrass near Kennerdell, on the line of the Allegheny Valley Railroad. Mr. George Fetterman one day found a can of nitro-glycerine hidden in the bushes by the roadside. When poured out of the can the stuff resembled lard oil. Mr. Fetterman owned several wells in the vicinity. Unconscious of its deadly contents, he took the can to his engine house, and for two weeks oiled his engine with nitro-glycerine. While putting out a well, one of the engines journals became heated. Fetterman picked up his oil can and oiled the heated part. The compound then exploded. Fetterman was dragged out of the ruins of the engine house still breathing, but he died in a few minutes.

The first victim who was blown to pieces while transporting the stuff to a well was Charles Clark. Loaded on his buckboard was his rack and four cans of nitro-glycerine. While driving over a rough road near Enterprise the glycerine exploded. Clark was annihilated, the buckboard was reduced to kindling wood, and the horse was blown into fragments. This was in August, 1871.

